# Performance Evaluation Model for Application Layer Firewalls

**DOI:** 10.1371/journal.pone.0167280

**Published:** 2016-11-28

**Authors:** Shichang Xuan, Wu Yang, Hui Dong, Jiangchuan Zhang

**Affiliations:** Information Security Research Center, Harbin Engineering University, Harbin, China; University of Texas at San Antonio, UNITED STATES

## Abstract

Application layer firewalls protect the trusted area network against information security risks. However, firewall performance may affect user experience. Therefore, performance analysis plays a significant role in the evaluation of application layer firewalls. This paper presents an analytic model of the application layer firewall, based on a system analysis to evaluate the capability of the firewall. In order to enable users to improve the performance of the application layer firewall with limited resources, resource allocation was evaluated to obtain the optimal resource allocation scheme in terms of throughput, delay, and packet loss rate. The proposed model employs the Erlangian queuing model to analyze the performance parameters of the system with regard to the three layers (network, transport, and application layers). Then, the analysis results of all the layers are combined to obtain the overall system performance indicators. A discrete event simulation method was used to evaluate the proposed model. Finally, limited service desk resources were allocated to obtain the values of the performance indicators under different resource allocation scenarios in order to determine the optimal allocation scheme. Under limited resource allocation, this scheme enables users to maximize the performance of the application layer firewall.

## Introduction

The current era of rapid internet technology development is witnessing widespread use of network communication in daily life, and hence, it is being increasingly influenced by internet security issues. Although users benefit significantly from the convenience afforded by internet technology, they are deeply concerned by their exposure to various network security risks. The need for achieving a trade-off between convenience and risk avoidance has led to the emergence of network security as an important issue. Consequently, a firewall has been introduced as a network security technology. A firewall is a rule engine that uses a collection of rules to match data packets with the rules in a rule matching process based on the order of the data packets. This is done until a matching rule is obtained, which determines the action to be applied to the corresponding packet as set forth by the rule.

Although application layer firewalls can provide comprehensive security, they have an adverse effect on network traffic processing performance. All traffic should be processed through the application layer firewall, so it is very likely to become the bottleneck of network communication and affect the user experience. In the firewall design and development process, a series of experiments is required to verify the system resource allocation in order to maximize the overall performance of the equipment. Although extensive testing is necessary, it is time-consuming and incurs high resource costs. If a mathematical model with a high degree of fit to application layer firewalls can be developed and used to analyze the key performance indicators of these firewalls, firewall developers can significantly reduce the testing time and developmental costs. Toward this end, the present article uses mathematical queuing theory as a basis to establish a performance evaluation model for application layer firewalls. The model is used to develop a resource allocation scheme with optimal performance indicators. Thus, it achieves the objective of effectively guiding firewall design.

The remainder of this paper is organized as follows. Section II reviews related studies and highlights our specific innovations. Section III presents the overall model and mathematical deductions. Section IV describes a simulation study of the model, in which resource allocation analysis is conducted with limited resources. Finally, Section V summarizes the study and concludes the paper.

## Related work

Firewall systems have been investigated for many years. Cyber threats are becoming more sophisticated, and the attack methods and frequency of attacks are increasing [1]. An application layer firewall mainly includes the analysis of the user behavior, rule-based detection, and defense against DDoS attacks [2]. Prokhorenko V et al. proposed a supervision framework and a web application protection model [3, 4]. Peng et al. discussed the related content of forensic authorship analysis [5]. They analyzed user profiling in intrusion detection [6] and they conducted a thorough study of astroturfing detection in media [7, 8]. Osanaiye et al. studied the defense against DDoS attack, and presented a taxonomy of the different types of cloud DDoS attacks, and the corresponding DDoS defense taxonomy [9]. In addition, they also proposed an Ensemble-based multi-filter feature selection method to detect DDoS attacks in cloud computing [10].

Several researchers have made important contributions to the development of firewall technology and optimization of network performance [11–20]. Some researchers have investigated the process of network packet acceptance in Linux or FreeBSD [21, 22], while others have adopted queuing theory to model systems more effectively [23–26]. Previous studies on modeling and analysis of network equipment performance have yielded some well-established results, especially in relation to findings based on queuing theory. Some studies have used the general queuing model (e.g., M/M /1, M/G/1, M/G/M/K, and Erlang’s formula) to capture and analyze the behaviors of cloud systems and applications [27–29]. Salah et al. studied the multi-service-desk queuing system [30]. This system consists of two service stages, the second of which involves multiple service desks. The model mainly evaluates the response time of cloud applications on the basis of performance indicators such as throughput, request loss probability, queuing probability, and CPU utilization. An extension of this model to a system with three service stages has been discussed, where both the second stage and the third stage involve multiple service desks [31, 32]. At the University of Electronic Science and Technology, China, Yang et al. established an M/M/m/m+r model to study the response time distribution of cloud service systems [33]. Similarly, Khazaei et al. modeled cloud computing centers using an M/G/m/m+r model, i.e., an approximate analytical model, to accurately estimate the complete probability distribution of the request response time and other important performance indicators [34].

Some key research articles have discussed the application of queuing theory to the analytical modeling of application layer firewalls and other security gateway devices. Salah, who conducted numerous studies in this area, obtained some remarkable results by applying queuing theory to firewall performance evaluation. In 2011, he proposed a two-stage queuing service system with different service rates in each stage [35]. His findings served as guidelines for performance analysis modeling based on queuing theory. However, the core firewall rules were not introduced into the above-mentioned model. Later, Salah et al. proposed a multi-stage queuing service system with the same service rate across all stages except the first stage [36, 37]. Similarly, in 2014, Salah used an Erlangian service model to describe a multi-stage queuing service system with the same service rate across all stages [38]. The above-mentioned studies have applied queuing theory at the rule level, which is more in line with the actual operation of firewalls. In 2015, Zapechnikov et al. proposed an analytical model based on the Erlangian model to study the performance of queuing systems with finite queues and multiple service stages. In relation to modern application layer firewalls that cover a variety of applications during application layer filtering, they constructed the second service stage as a hyper-Erlangian queuing model [39].

In summary, the following issues have been raised by previous studies. First, models established on the basis of a single service layer lack a comprehensive representation of the system. Second, the service process in a single layer does not incorporate a rule engine; instead, it is treated as a single process. Third, some studies have assumed a single-service desk model. As current system hardware usually supports multi-core processors, this assumption is not realistic. Fourth, for convenience of deduction, the average value of the time parameter is used, while the randomness of probability events is overlooked. The present article addresses the aforementioned issues and establishes a rule-based multi-service-window, multi-layer model. In addition, system performance is analyzed from the perspective of resource allocation.

## Model Analysis

In this study, we mainly discuss rule-based detection mode. A multi-service-desk, multi-layer model is combined with a rule-matching Erlangian model to establish an accurate description of the application layer firewall (ALF) model.

**Fig 1:**
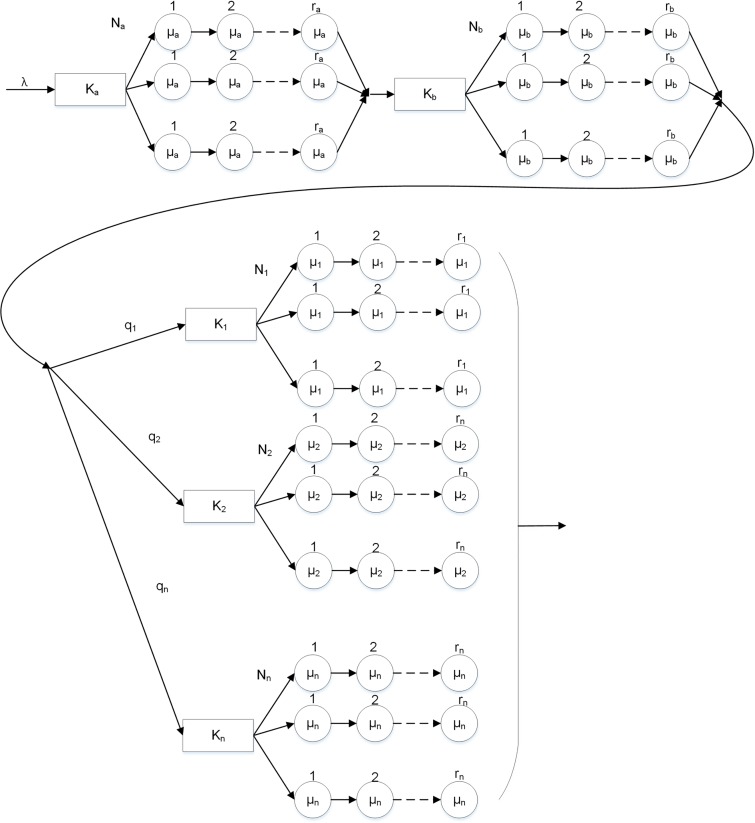
Application layer firewall (ALF) model.

The ALF model is a multi-service-desk, three-layer queuing model whose service time follows the Erlang distribution. In the model, a data packet is first processed by the network and transport layers. Once the data packet arrives at the application layer, it can join different application layer queues depending on the previously processed results. The ALF model is shown in Fig 1.

The model parameters are defined as follows. The packet arrival rate of the system is denoted by λ. In the first layer, i.e., the network layer, K_a_ is the buffer queue capacity, N_a_ is the number of service windows, r_a_ is the number of rules, and μ_a_ is the service rate. In the second layer, i.e., the transport layer, K_b_ is the buffer queue capacity, N_b_ is the number of service windows, r_b_ is the number of rules, and μ_b_ is the service rate. In the third layer, i.e., the application layer, K_1_, K_2_,…, K_n_ are the buffer queue capacities, N_1_, N_2_,…, N_n_ are the number of service windows, r_1_, r_2_,…, r_n_ are the number of rules, and μ_1_, μ_2_,. . .. . ., μ_n_ are the service rates of application 1, 2, …, n, respectively. Further, q_1_, q_2_,…, q_n_ are the probabilities that an arrived packet belongs to application 1, 2, …, n, respectively.

In terms of the basic multi-layer model, the ALF model further divides the service process of each layer into several continuous service stages. In the application layer, it corrects and improves the construction of multiple applications of the WEB-EG model. Therefore, parallel processing of all the service desks of different applications is achieved along with optimization of service desk utilization in the application layer.

### Analysis of single layer

**Fig 2:**
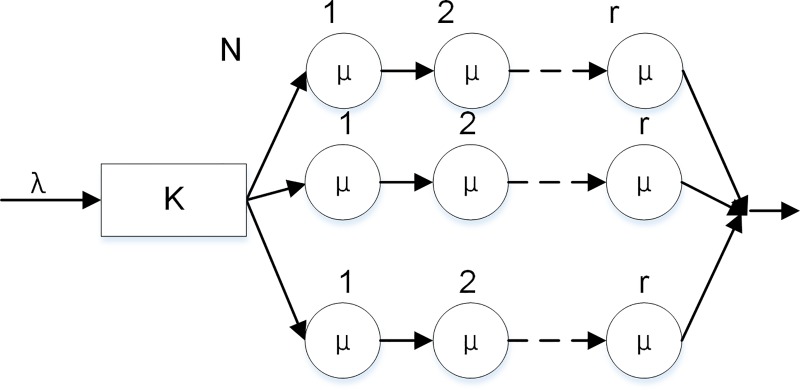
Single layer of the ALF model.

In the deduction process of the multi-layer model, we first deduct each of the three layers of the system. Then, the deductions are combined for the overall analysis. The modeling of various applications in the network, transport, and application layers is shown in Fig 2.

There are some processing differences between a multi-service-window Erlang queuing system and a single-service-desk M/E_k_/1/K model. The multi-service-window model can be converted into equivalent single-service-desk models. In the system, there are N service desks servicing at the same time, each with service rate μ. Thus, the total service rate of the system is Nμ. The maximum capacity of the system is the sum of the buffer queue capacities and number of service windows, i.e., K+N. The M/G/1/K analytical method is used to analyze the obtained equivalent M/E_k_/1/K model. The single-service-desk model assumes that data packets arrive according to a Poisson process with parameter λ. The buffer queue capacity is K + N-1, the number of rules is r, the service time has an Erlang distribution, and the service rate is Nμ. Therefore, the service time distribution density function is given by

$$f\left(t\right)=\frac{N\;\mu {\left(N\;\mu t\right)}^{r-1}}{\left(r-1\right)!}e^{-N\;\mu t}\;\left(t\geq 0\right)$$ (1)

Further, *α*_*k*_ is the probability that k packets arrive at the system during the service time of a packet. Therefore,

$$\partial_{k}=\int_{0}^{\infty }\frac{{\left(\lambda x\right)}^{k}}{k!}e^{-\lambda x}\;f\left(x\right)dx$$ (2)

Substituting (1) into (2) gives

$$\partial_{k}=\frac{\lambda^{k}}{k!}\frac{{\left(N\;\mu \right)}^{r}}{\left(r-1\right)!}\int_{0}^{\infty }x^{k+r-1}e^{-\left(\lambda +N\;\mu \right)x}dx$$ (3)

From the gamma function formula,

$$\int_{0}^{\infty }x^{s-1}e^{-\lambda x}dx=\frac{\Gamma \left(s\right)}{\lambda^{s}}$$ (4)

Therefore, the following relationship is obtained:

$$\partial_{k}=\frac{\lambda^{k}}{k!}\frac{{\left(N\;\mu \right)}^{r}}{\left(r-1\right)!}\cdot \frac{\Gamma \left(k+r\right)}{{\left(\lambda +N\;\mu \right)}^{k+r}}=\frac{\lambda^{k}}{k!}\frac{{\left(N\;\mu \right)}^{r}}{\left(r-1\right)!}\cdot \frac{\left(k+r-1\right)!}{{\left(\lambda +N\;\mu \right)}^{k+r}}$$ (5)

At the instant when a packet leaves, the number of packets in the system lies in the range [0, K + N-1]. At any other given time, the number of packets in the system lies in the range [0, K + N]. In the Markov process, the state transition probability P_jk_ denotes the probability of j number of packets changed to k number at any given time within the system. The state transition is related to the number of packets arriving within the service time. Therefore, the relationship between P_jk_ and α_k_ is given by

$$p_{0k}=\left\{ \begin{array}{l} \partial_{k}\;0\leq k\leq K+N-2\\ \sum_{m=K+N-1}^{\infty }\partial_{m}\;k=K+N-1 \end{array}\right.\;j=0$$ (6)

$$p_{jk}=\left\{ \begin{array}{l} \partial_{k-j+1}\;j-1\leq k\leq K+N-2\\ \sum_{m=K+N-1}^{\infty }\partial_{m}\;k=K+N-1 \end{array}\right.\;1\leq j\leq K+N-1$$ (7)

At the instant when a packet leaves, the system’s steady state probability is π_k_ (0≤k≤K + N-1), and the following relationship exists between different states:

$$\pi_{k}=\sum_{j=0}^{K-1}\pi_{j}p_{jk}\;0\leq k\leq K+N-1$$ (8)

From (6), (7), and (8), it can be deduced that

$$\pi_{k}=\pi_{0}\partial_{k}+\sum_{j=1}^{k+1}\pi_{j}\partial_{k-j+1}\;0\leq k\leq K+N-2$$ (9)

Further, π_0_ is used in the following calculation:

$$\frac{\pi_{k+1}}{\pi_{0}}=\frac{1}{\partial_{0}}\left(\frac{\pi_{k}}{\pi_{0}}-\sum_{j=1}^{k}\frac{\pi_{j}}{\pi_{0}}\partial_{k-j+1}-\partial_{k}\right)\;0\leq k\leq K+N-2$$ (10)

In accordance with the regularity conditions,

$$\sum_{k=0}^{K+N-1}\pi_{k}=1$$ (11)

From (10) and (11), the value of π_0_ can be obtained as

$$\pi_{0}=\frac{1}{1+\sum_{k=1}^{K-1}\frac{\pi_{k}}{\pi_{0}}}$$ (12)

Further, P_k_ denotes the probability that k data packets exist in the system at any given time, and P_loss_ denotes the packet loss rate, where packet loss occurs owing the arrival of packets at a full queue.

The system throughput, denoted by the packet departure rate, can be expressed as

$$\gamma =\frac{1-p_{0}}{\bar{X}}=\frac{1-p_{0}}{r}\cdot \mu$$ (13)

In addition,

$$\gamma =\frac{1-p_{0}}{r}\cdot \mu =\lambda \left(1-p_{loss}\right)$$ (14)

The following relationship can be obtained:

$$p_{loss}=p_{K}=1-\frac{1-p_{0}}{\rho }=\frac{p_{0}+\rho -1}{\rho }$$ (15)

where $\rho =\lambda \bar{X}$ denotes the offered load of the system. Finally, the packet loss rate can be deduced as follows:

$$p_{loss}=p_{K}=1-\frac{1}{\pi_{0}+\rho }$$ (16)

The average number of packets in the system is given by

$$\bar{K}=\sum_{k=1}^{K}kp_{k}=\frac{1}{\pi_{0}+\rho }\sum_{k=0}^{K-1}k\pi_{k}+K\left(1-\frac{1}{\pi_{0}+\rho }\right)$$ (17)

The average time spent by packets in the system is given by

$$\bar{W}=\frac{\bar{K}}{\gamma }=\frac{1}{\lambda }\left(\sum_{k=0}^{K-1}k\pi_{k}+K\left(1-\frac{1}{\pi_{0}+\rho }\right)\right)$$ (18)

Finally, the average queuing time is obtained as

$$W_{q}=\bar{W}-\bar{X}=\frac{1}{\lambda }\left(\sum_{k=0}^{K-1}k\pi_{k}+K\left(\pi_{0}+\rho -1\right)-\rho \right)$$ (19)

In accordance with this method, deduction was carried out for each layer of the queuing system. The layers were combined to perform the overall analysis of the system.

### Analysis of the first layer

In the first layer, the data packet arrival rate is the overall system arrival rate λ. The buffer capacity is K_a_, the number of service windows is N_a_, the number of rules is r_a_, and the service rate is μ_a_.

In this Erlangian service queuing model, the probability that k packets arrive at the queue during the service time of a packet is given by

$$\begin{array}{l} \partial_{k}=\frac{\lambda^{k}}{k!}\frac{{\left(N_{a}\mu_{a}\right)}^{r}}{\left(r_{a}-1\right)!}\cdot \frac{\left(k+r_{a}-1\right)!}{{\left(\lambda +N_{a}\mu_{a}\right)}^{k+r_{a}}} \end{array}$$ (20)

The probability of state transition in the Markov process is given by

$$p_{0k}=\left\{ \begin{array}{l} \partial_{k}\;0\leq k\leq K_{a}+N_{a}-2\\ \sum_{m=K_{a}+N_{a}-1}^{\infty }\partial_{m}\;k=K_{a}+N_{a}-1 \end{array}\right.\;j=0$$ (21)

$$p_{jk}=\left\{ \begin{array}{l} \partial_{k-j+1}\;j-1\leq k\leq K_{a}+N_{a}-2\\ \sum_{m=K_{a}+N_{a}-1}^{\infty }\partial_{m}\;k=K_{a}+N_{a}-1 \end{array}\right.\;1\leq j\leq K_{a}+N_{a}-1$$ (22)

At the instant when a packet leaves, the system’s steady state probability is given by

$$\pi_{k}=\sum_{j=0}^{K_{a}+N_{a}-1}\pi_{j}p_{jk}\;0\leq k\leq K_{a}+N_{a}-1$$ (23)

Further, π_0_ is used in the following calculation:

$$\frac{\pi_{k+1}}{\pi_{0}}=\frac{1}{\partial_{0}}\left(\frac{\pi_{k}}{\pi_{0}}-\sum_{j=1}^{k}\frac{\pi_{j}}{\pi_{0}}\partial_{k-j+1}-\partial_{k}\right)\;0\leq k\leq K_{a}+N_{a}-2$$ (24)

The value of π_0_ is obtained under regularity conditions as

$$\pi_{0}=\frac{1}{1+\sum_{k=1}^{K_{a}+N_{a}-1}\frac{\pi_{k}}{\pi_{0}}}$$ (25)

The offered load of the network layer is given by

$$\rho =\lambda \cdot \frac{r_{a}}{N_{a}\mu_{a}}$$ (26)

The packet loss rate for this layer’s queuing system is obtained as

$$p_{loss,a}=1-\frac{1}{\pi_{0}+\rho }$$ (27)

Therefore, the throughput of this layer is given by

$$\gamma_{a}=\lambda \left(1-p_{loss,a}\right)$$ (28)

The average queuing time of packets in this layer is given by

$$W_{q,a}=\frac{1}{\lambda }\left(\sum_{k=0}^{K_{a}+N_{a}-1}k\pi_{k}+K_{a}\left(\pi_{0}+\rho -1\right)-\rho \right)$$ (29)

### Analysis of the second layer

In the second layer, the data packet arrival rate is the throughput γ_a_ of the first layer, i.e., the network layer. The buffer capacity is K_b_, the number of service windows is N_b_, the number of rules is r_b_, and the service rate is μ_b_.

As with the analysis of the first layer, the probability of k packets arriving at the queue during the service time of a packet is given by

$$\partial_{k}=\frac{\gamma_{a}{}^{k}}{k!}\frac{{\left(N_{b}\mu_{b}\right)}^{r}}{\left(r_{b}-1\right)!}\cdot \frac{\left(k+r_{b}-1\right)!}{{\left(\lambda +N_{b}\mu_{b}\right)}^{k+r_{b}}}$$ (30)

The probability of state transition in the Markov process is given by

$$p_{0k}=\left\{ \begin{array}{l} \partial_{k}\;0\leq k\leq K_{b}+N_{b}-2\\ \sum_{m=K_{b}+N_{b}-1}^{\infty }\partial_{m}\;k=K_{b}+N_{b}-1 \end{array}\right.\;j=0$$ (31)

$$p_{jk}=\left\{ \begin{array}{l} \partial_{k-j+1}\;j-1\leq k\leq K_{b}+N_{b}-2\\ \sum_{m=K_{b}+N_{b}-1}^{\infty }\partial_{m}\;k=K_{b}+N_{b}-1 \end{array}\right.\;1\leq j\leq K_{b}+N_{b}-1$$ (32)

At the instant when a packet leaves, the system’s steady state probability is given by

$$\pi_{k}=\sum_{j=0}^{K_{b}+N_{b}-1}\pi_{j}p_{jk}\;0\leq k\leq K_{b}+N_{b}-1$$ (33)

Further, π_0_ is used in the following calculation:

$$\frac{\pi_{k+1}}{\pi_{0}}=\frac{1}{\partial_{0}}\left(\frac{\pi_{k}}{\pi_{0}}-\sum_{j=1}^{k}\frac{\pi_{j}}{\pi_{0}}\partial_{k-j+1}-\partial_{k}\right)\;0\leq k\leq K_{b}+N_{b}-2$$ (34)

The value of π_0_ is obtained under regularity conditions as

$$\pi_{0}=\frac{1}{1+\sum_{k=1}^{K_{b}+N_{b}-1}\frac{\pi_{k}}{\pi_{0}}}$$ (35)

The offered load of the transport layer is given by

$$\rho =\gamma_{a}\cdot \frac{r_{b}}{N_{b}\mu_{b}}$$ (36)

The packet loss rate for this layer’s queuing system is obtained as

$$p_{loss,b}=1-\frac{1}{\pi_{0}+\rho }$$ (37)

Therefore, the throughput of this layer is given by

$$\gamma_{b}=\gamma_{a}\left(1-p_{loss,b}\right)$$ (38)

The average queuing time of packets in this layer is given by

$$W_{q,b}=\frac{1}{\lambda }\left(\sum_{k=0}^{K_{b}+N_{b}-1}k\pi_{k}+K_{b}\left(\pi_{0}+\rho -1\right)-\rho \right)$$ (39)

### Analysis of the third layer

In the third layer, the data packet arrival rate is the throughput γ_b_ of the second layer, i.e., the transport layer. In the application layer, K_1_, K_2_,…, K_n_ are the buffer capacities, N_1_, N_2_,…, N_n_ are the number of service windows, r_1_, r_2_,…, r_n_ are the number of rules, and μ_1_, μ_2_,…, μ_n_ are the service rates of application 1, 2, …, n, respectively. Further, q_1_, q_2_, …, q_n_ are the probabilities that an arrived packet belongs to application 1, 2, …, n, respectively, while q_1_γ_b_, q_2_γ_b_, …, q_n_γ_b_ are the respective arrival rates of the packets.

For the queuing system that processes a packet belonging to application *i*, the probability that k data packets arrive at the queue during the service time is given by

$$\partial_{k}=\frac{{\left(q_{i}\gamma_{b}\right)}^{k}}{k!}\frac{{\left(N_{i}\mu_{i}\right)}^{r}}{\left(r_{i}-1\right)!}\cdot \frac{\left(k+r_{i}-1\right)!}{{\left(\lambda +N_{i}\mu_{i}\right)}^{k+r_{i}}}$$ (40)

The relationship between the steady state probabilities is given by

$$\frac{\pi_{k+1}}{\pi_{0}}=\frac{1}{\partial_{0}}\left(\frac{\pi_{k}}{\pi_{0}}-\sum_{j=1}^{k}\frac{\pi_{j}}{\pi_{0}}\partial_{k-j+1}-\partial_{k}\right)\;0\leq k\leq K_{i}+N_{i}-2$$ (41)

The value of π_0_ is obtained under regularity conditions as

$$\pi_{0}=\frac{1}{1+\sum_{k=1}^{K_{i}+N_{i}-1}\frac{\pi_{k}}{\pi_{0}}}$$ (42)

In the application layer, the offered load of the packet that enters application *i* is given by

$$\rho =q_{i}\gamma_{b}\cdot \frac{r_{i}}{N_{i}\mu_{i}}$$ (43)

The packet loss rate of this application in the application layer is obtained as

$$p_{loss,i}=1-\frac{1}{\pi_{0}+\rho }$$ (44)

Thus, the throughput of data entering the application is given by

$$\gamma_{i}=q_{i}\gamma_{b}\left(1-p_{loss,i}\right)$$ (45)

The average queuing time spent by the data packet entering application *i* in the application layer is given by

$$W_{q,i}=\frac{1}{\lambda }\left(\sum_{k=0}^{K_{i}+N_{i}-1}k\pi_{k}+K_{i}\left(\pi_{0}+\rho -1\right)-\rho \right)$$ (46)

For the entire queuing system, the total throughput is the data output rate of the last layer. Here, the total throughput is represented as the throughput of all the applications in the application layer, i.e.,

$$\gamma =\sum_{i=1}^{n}q_{i}\gamma_{b}\left(1-p_{loss,i}\right)$$ (47)

The packet loss rate of the next layer is the proportion of packets that have left the system owing to full buffer queues in the previous layer. Then, for a specific layer, the proportion of packets lost in the overall system can be obtained by multiplying the packet loss rate of the previous layer with that of the current layer. The average packet loss rate of the application layer is given by

$$p_{loss,c}=\sum_{i=1}^{n}q_{i}\cdot p_{loss,i}$$ (48)

Therefore, the overall packet loss rate of the system is given by

$$p_{loss}=p_{loss,a}+\left(1-p_{loss,a}\right)p_{loss,b}+\left[1-p_{loss,a}-\left(1-p_{loss,a}\right)p_{loss,b}\right]p_{loss,c}$$ (49)

In the application layer, the average queuing time is given by

$$W_{q,c}=\sum_{i=1}^{n}q_{i}\cdot W_{q,i}$$ (50)

Therefore, it is possible to obtain the average queuing time of packets from the network layer to the application layer as follows:

$$W_{q}=W_{q,a}+W_{q,b}+W_{q,c}=W_{q,a}+W_{q,b}+\sum_{i=1}^{n}q_{i}\cdot W_{q,i}$$ (51)

## Experimental Evaluation

This section describes a discrete event simulation method used to validate the ALF model. The basic principle is to use computer simulation to simulate discrete event systems.

In accordance with the performance evaluation model designed in this study, during the experimental procedure, event arrival was modeled as a Poisson process. The service model was designed as an Erlangian service process. Further, the experimental parameters were set to obtain the required results. Although the arrival time and service time of events were randomly generated, the time distribution was not even. Therefore, an exponential distribution was generated using Erlang-distributed random numbers.

In terms of the arrival time, the following expression should be used to obtain the exponentially distributed random numbers.

$$T_{i}=-\frac{1}{\lambda }\cdot \ln\left(rand_{0,1}\right)$$ (52)

where *rand*_0,1_ is a uniformly distributed random number in the range (0,1), which is generated by the simulation process, *T*_i_ is the exponentially distributed random number required in the simulation process, and λ is the parameter of the exponential distribution in the model. The exponentially distributed random number was applied to the random generation of data packet arrival time intervals.

In terms of the Erlang-distributed random numbers, the generation method was similar to that used for exponentially distributed random numbers. For the k^th^ stage of the Erlang distribution, the expression for generating the random value of the service time is given by

$$T_{s}=-\frac{1}{\mu }\cdot \ln\left(\prod_{i=1}^{k}rand_{0,1}\right)$$ (53)

where it is necessary to continuously generate k random values for k service stages, T_s_ is the random value of the Erlang-distributed total service time, and μ is the parameter of the Erlang distribution in the k^th^ stage, i.e., the service time of each stage is subjected to a negative exponential distribution with parameter μ. The Erlang-distributed random numbers were applied to the rule-based service matching process to obtain the total service time.

The performance of the system model was evaluated under different conditions of CPU resource allocation. It was necessary to list all the different resource allocation combinations given the total resources available, which were then input to the theoretical formula and simulation program for the calculation of throughput.

### Experiment 1

There were 6 service desks and 2 applications. The probabilities that a data packet belonged to application layer 1 and application layer 2 were q_1_ = 50% and q_2_ = 50%, respectively. The packet arrival rate was λ = 200 kpps (1000 packets per second). The processing rates of the network layer, transport layer, and two application layers were μ_a_ = 250 kpps, μ_b_ = 333 kpps, μ_1_ = 145 kpps, and μ_2_ = 180 kpps, respectively. The buffer capacities of the network layer, transport layer, and two application layers were K_a_ = 100, K_b_ = 50, and K_1_ = 100, and K_2_ = 50, respectively. The number of rules for the network layer, transport layer, and two application layers were r_a_ = 5, r_b_ = 5, r_1_ = 5, and r_2_ = 5, respectively. The test results are listed in Table 1.

**Table 1:** Test results of Experiment 1.

Resource allocation conditions				Throughput (kpps)				Packet loss rate (%)			
N_a_	N_b_	N_1_	N_2_	1	2	3	Mean	1	2	3	Mean
3	1	1	1	62.03	62.26	62.01	62.10	68.50	68.71	68.33	68.51
2	2	1	1	64.97	65.06	64.65	64.89	67.64	67.74	67.68	67.69
1	3	1	1	48.69	50.16	49.65	49.5	75.17	75.31	74.90	75.13
2	1	2	1	65.98	66.34	66.40	66.24	66.76	66.81	66.39	66.65
1	2	2	1	49.47	49.87	48.89	49.41	74.91	75.06	75.41	75.13
1	1	3	1	50.01	49.85	50.05	49.97	74.89	75.23	75.11	75.08
2	1	1	2	61.99	62.14	62.18	62.10	68.58	68.74	68.44	68.59
1	2	1	2	50.11	49.85	49.33	49.76	75.17	74.82	75.27	75.09
1	1	2	2	49.49	49.87	49.97	49.78	74.76	75.04	74.83	74.88
1	1	1	3	50.03	49.86	49.24	49.71	75.13	75.17	75.21	75.17

The experimental results showed that the value of the throughput was maximized when resource allocation was specified as N_a_ = 2, N_b_ = 1, N_1_ = 2, and N_2_ = 1. These results were consistent with the theoretical results.

### Experiment 2

There were 5 service desks and 2 applications. The probabilities that a data packet belonged to application layer 1 and application layer 2 were q_1_ = 50% and q_2_ = 50%, respectively. The packet arrival rate was λ = 150 kpps (1000 packets per second). The processing rates of the network layer, transport layer, and two application layers were μ_a_ = 500 kpps, μ_b_ = 250 kpps, μ_1_ = 333 kpps, ands μ_2_ = 500 kpps, respectively. The buffer capacities of the network layer, transport layer, and two application layers were K_a_ = 100, K_b_ = 100, K_1_ = 10, and K_2_ = 10, respectively. The number of rules for the network layer, transport layer, and two application layers were r_a_ = 5, r_b_ = 3, r_1_ = 5, and r_2_ = 5, respectively. The test results are listed in Table 2.

**Table 2:** Test results of Experiment 2.

Resource allocation conditions				Throughput (kpps)				Packet loss rate (%)			
N_a_	N_b_	N_1_	N_2_	1	2	3	Mean	1	2	3	Mean
2	1	1	1	82.32	82.78	82.97	82.69	44.51	44.40	44.57	44.49
1	2	1	1	98.75	99.14	98.98	98.96	33.29	33.05	33.41	33.25
1	1	2	1	82.46	83.01	82.17	82.55	44.44	44.63	44.55	44.53
1	1	1	2	82.35	82.79	82.26	82.47	44.50	44.56	44.47	44.51

The experimental results showed that the value of the throughput was maximized when the resource allocation was specified as N_a_ = 1, N_b_ = 2, N_1_ = 1, and N_2_ = 1. These results were consistent with the theoretical results.

## Conclusion

On the basis of previous studies, the present article established a complex performance evaluation model for application firewalls that is based on an Erlangian multi-service-desk model with three service layers, namely the ALF model. Theoretical analysis and deductions were carried out using this model. We derived the theoretical throughput, packet loss ratio, and average delay. We started from the basic model constituting the overall system and demonstrated the deduction process for a single-layer queuing system based on an Erlang multi-service-desk model with mixed layers. Then, the overall system analysis was carried out to account for the ALF’s multi-layer structure and the different types of applications in the application layer. System performance indicators, such as packet loss rate, throughput, and average queue time, were obtained. Finally, experimental evaluations were carried out to compare the theoretical and experimental values of the performance indicators under different resource allocation schemes for the ALF model. During the model establishment and analysis process, multi-service-desk allocation scenarios were fully considered. Thus, the number of service desks in each layer was involved in the calculation of each performance indicator. The experimental results showed that the allocation of CPU resources can directly influence the overall performance of application layer firewall systems. Moreover, a reasonable allocation of resources can effectively improve the performance of application layer firewall. Therefore, this model can be used as a reference for the design of application layer firewall. In the future, we will extend our work to include the analysis of the user behavior, throttling the number of connections, and DDoS detection.
